# Proposal for a new mediastinal compartment classification of transverse plane images according to the Japanese Association for Research on the Thymus (JART) General Rules for the Study of Mediastinal Tumors

**DOI:** 10.3892/or.2013.2904

**Published:** 2013-12-06

**Authors:** KIMINORI FUJIMOTO, MASAKI HARA, NORIYUKI TOMIYAMA, MASAHIKO KUSUMOTO, FUMIKAZU SAKAI, YOSHITAKA FUJII

**Affiliations:** 1 Department of Radiology, Kurume University School of Medicine, and Center for Diagnostic Imaging, Kurume University Hospital, Kurume, Fukuoka 830-0011, Japan; 2 Department of Radiology, Nagoya City West Medical Center, Kita-ku, Nagoya 462-8508, Japan; 3 Department of Radiology, Osaka University Graduate School of Medicine, Suita, Osaka 565-0871, Japan; 4 Department of Diagnostic Radiology, National Cancer Center Hospital, Chuo-ku, Tokyo 104-0045, Japan; 5 Department of Diagnostic Radiology, Saitama International Medical Center, Saitama Medical University, Hidaka, Saitama 350-1298, Japan; 6 Department of Oncology Immunology and Surgery, Nagoya City University Graduate School of Medical Sciences, Nagoya 467-8601, Japan

**Keywords:** mediastinum, compartment, mediastinal mass, mediastinal neoplasm, computed tomography, differential diagnosis

## Abstract

There is no existing worldwide published method for mediastinum compartment classification based on transverse section images for the differential diagnosis of mediastinal tumors. Herein, we describe a new method for anatomic mediastinal compartment classification using transverse section computed tomography (CT) images and the use of this method to classify mediastinal lesions, and thus evaluate whether the method is sufficiently user-friendly and useful. In a publication of the Japanese Association for Research on the Thymus (JART), we proposed the following four mediastinal compartments based on transverse CT images: superior portion of mediastinum, anterior mediastinum (prevascular zone), middle mediastinum (peri-tracheoesophageal zone), and posterior mediastinum (paravertebral zone). In the present study, we retrospectively analyzed 445 pathologically proven mediastinal mass lesions, and categorized them into the proposed four compartments by consensus reading. Mass lesions were classified into compartments based on the location of the lesion centroid, and each lesion was satisfactorily categorized into a compartment. Almost all thymic epithelial tumors (99%, 244/246), all 24 thymic malignant lymphomas and a majority of germ cell neoplasms (93%, 54/58) were classified as being in the anterior mediastinum compartment. The majority of intrathoracic goiters (82%, 14/17) were categorized as being in the superior portion of the mediastinum compartment. Approximately two-thirds of mass lesions in the middle mediastinum were cysts, including foregut and pericardial cysts. Approximately 80% of 37 mass lesions in the posterior mediastinum were neurogenic tumors. Correspondingly, 29 of the 49 neurogenic tumors (60%) were categorized as being in the posterior mediastinum, while 10 (20%) were in the superior portion of the mediastinum, 4 (8%) in the anterior mediastinum, and 6 (12%) in the middle mediastinum. Our findings showed that the newly proposed mediastinal compartment classification using transverse images appears to be user-friendly enough for practical clinical application and may be helpful in differential diagnoses.

## Introduction

The mediastinum constitutes a compartmentalized septum or partition that vertically divides the thorax (1). It is anatomically bound on the lateral side by the parietal pleural reflections along the medial aspects of both lungs, superiorly by the thoracic inlet, inferiorly by the diaphragm, anteriorly by the sternum, and posteriorly by the anterior surfaces of the thoracic vertebral bodies (1–3). When mediastinal mass lesions are diagnosed using imaging techniques, image interpretation requires accurate assessment of the lesion origin, area of existence and extension, and inner structures. Therefore, it is clinically important to have a standardized method for classifying the mediastinum into several compartments for the purpose of tumor description and categorization in differential diagnosis.

Published methods for classifying mediastinal compartments include the traditional method, Fraser and Paré method, Felson method, Heitzman method, Zylak method, and Whitten method (1–7). However, what is confusing is the fact that different authors use different terms and methods for the same thing. Among these methods the classification of the mediastinum into three compartments by Felson (4) is strictly based on radiology. This method is practical and user-friendly; however, it utilizes a lateral chest radiograph, which cannot distinguish between certain situations. For example, the anterior compartment can include some masses that are in both the anterior and middle mediastinum, and the middle compartment can include some masses that are both in the middle and posterior mediastinum.

X-ray computed tomography (CT) is currently the main practical clinical examination used for assessing the origin, existence, and extension of a lesion in complicated mediastinal structures (8–12). Thus, the mediastinum compartment needs to be classified based on the transverse plane image.

Our group of diagnostic radiologists from the Japanese Association for Research on the Thymus (JART) described a new method for classifying the mediastinal compartment using the transverse plane image in a recent set of Japanese General Rules for the Study of Mediastinal Tumors (13).

The purpose of this study was two-fold: firstly, to present the new method for anatomic mediastinal compartment classification using transaxial section images acquired by CT; and secondly, to assess whether the proposed method was user-friendly and applicable by using it to retrospectively classify a large number of mediastinal lesions.

## Materials and methods

The Ethics Committee of Kurume University approved this retrospective study (research no. 12002) and waived the requirement to obtain patient approval or informed consent for the retrospective review of their records and images. All research was in compliance with the principles of the Declaration of Helsinki (version 2008) of the World Medical Association.

**Figure 1:**
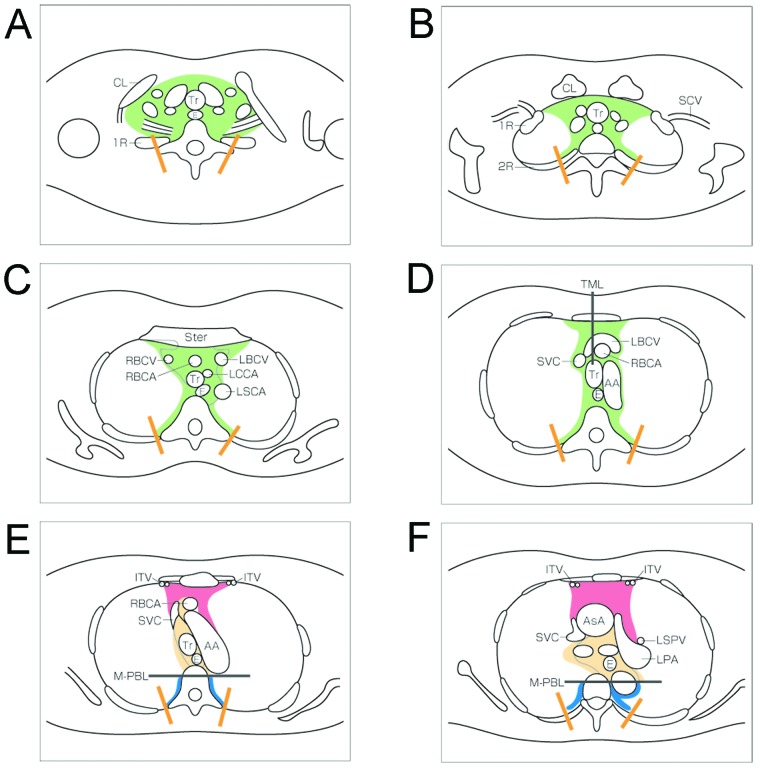

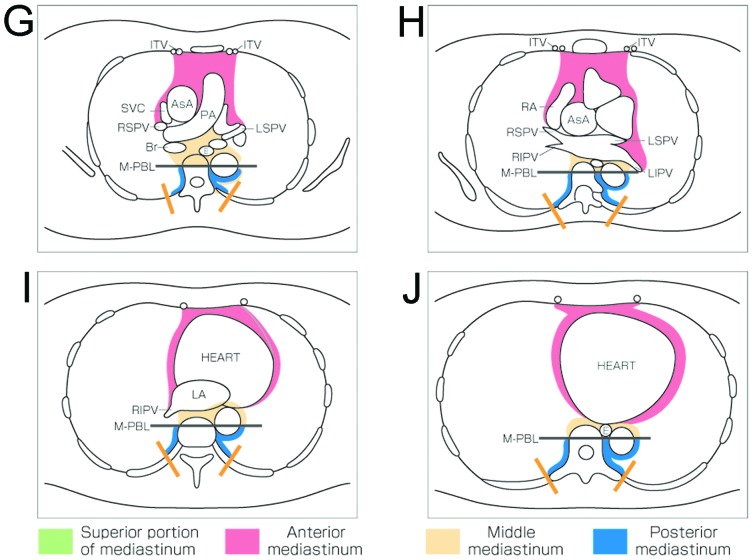
Schematic diagram represents the new proposal for mediastinal compartment classification according to the General Rules for Study of Mediastinal Tumors of the Japan Association for Research on the Thymus (JART). (A) Thoracic inlet, (B) upper rim of clavicle, (C) sterno-clavicular joint, (D) left brachiocephalic vein across TML, (E) aortic arch, (F) tracheal carina, (G) right main pulmonary artery, (H) pulmonary trunk, (I) left atrium, (J) tricuspid valve. Abbreviations: CL, clavicle; Tr, trachea; SCA, subclavian artery; SCV, subclavian vein; 1R, first rib; 2R, second rib; Ster, sternum; E, esophagus; RBCV, right brachicephalic vein; RBCA, right brachiocephalic artery; LBCV, left brachicephalic vein; LCCA, left common carotid artery; LSCA, left subclavian artery; SVC, superior vena cava; AA, aortic arch; AsA, ascending aorta; LPA, left pulmonary artery; ITV, internal thoracic vessels; Br, bronchus; LSPV, left superior pulmonary vein; RSPV, right superior pulmonary vein; PA, pulmonary artery; RA, right atrium; RIPV, right inferior pulmonary vein; LIPV, left inferior pulmonary vein; LA, left atrium; TML, tracheal mid-line; M-PBL, middle-posterior boundary line (see Materials and methods).

**Figure 2:**
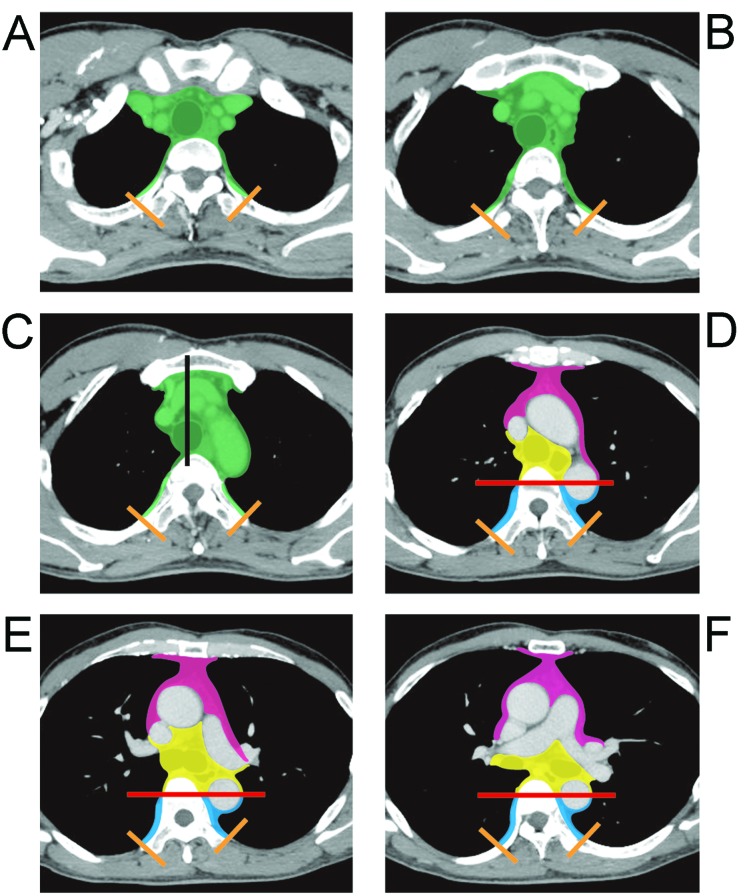

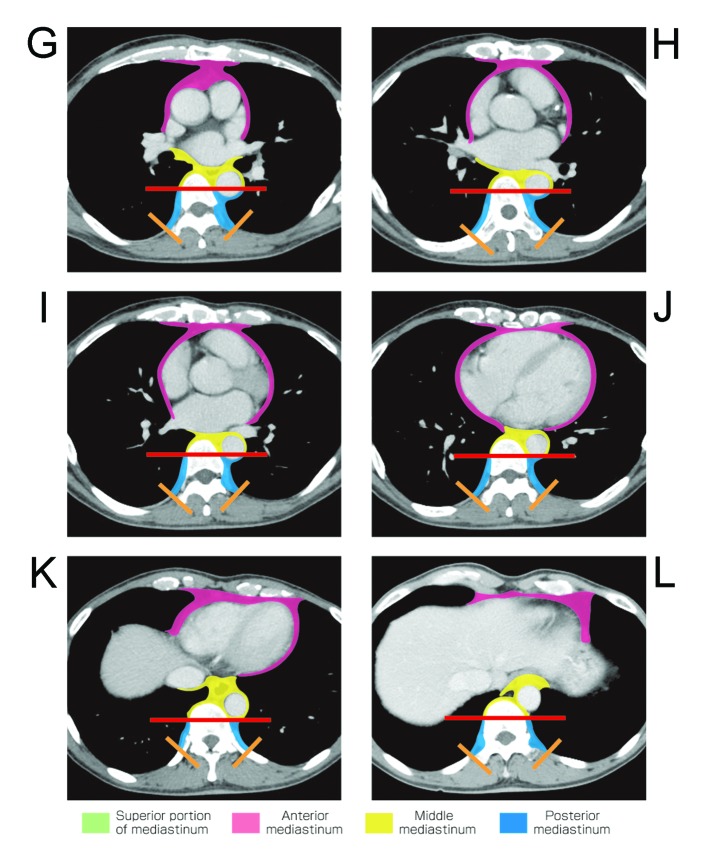
Contrast-enhanced CT images represent new proposal of mediastinal compartment classification according to the General Rules for Study of Mediastinal Tumors of the JART. (A) Thoracic inlet, (B) upper rim of clavicle, (C) sterno-clavicular joint, (D) left brachiocephalic vein across TML, (E) aortic arch, (F) tracheal carina, (G) right main pulmonary artery, (H) pulmonary trunk, (I) left atrium, (J) tricuspid valve, (K) hepatic dome of diaphragm, (L) middle of 12th thoracic vertebral body. Abbreviations: TML, tracheal mid-line; M-PBL, middle-posterior boundaryline (see Materials and methods).

We, five radiologists of the Diagnostic Imaging Committee of the JART, proposed the division of the mediastinum into the following four compartments that are visible on transverse CT images: superior portion of mediastinum, anterior mediastinum (prevascular zone), middle mediastinum (peri-tracheoesophageal zone), and posterior mediastinum (paravertebral zone) (13). The fundamental basis of this methodology is that a mass lesion is classified into one of the four compartments of which the arising organ or tissue of a mediastinal mass lesion exists and there is a potential space which a lesion easily extends. The anatomical structure and the virtual line, which are easy to identify during CT image interpretation and operation, were set as the index of the boundary. Table I and Figs. 1 and 2 show the details of the classification method.

From the end of December 2007, each of the five radiologists checked the medical records retrospectively and collected respectively 100 consecutive cases with a mediastinal mass that was proven surgically and/or pathologically in respective five institutions; thus 500 cases were collected in total. For each case, each radiologist selected at least one representative CT image that included the center of a mediastinal lesion, defined as the geometric center (centroid) in the transverse section showing the greatest size of the lesion.

Each independent radiologist evaluated each mediastinal lesion based on the CT image, and assigned each lesion to one of the proposed four compartments in individual institution. We further discussed whether each mediastinal lesion could be satisfactorily categorized into one of the four compartments without any contradictions, and whether this method was helpful in differential diagnosis.

## Results

Of the 500 selected cases, 55 were excluded for technical reasons, such as small size, insufficient image resolution and motion artifact. The remaining 445 cases were considered to be subjects. They included 246 thymic epithelial tumors (193 thymomas and 53 thymic carcinomas), 24 thymic malignant lymphomas, 31 malignant germ cell tumors, 27 mature teratomas, 49 neurogenic tumors, 17 intrathoracic goiters and 51 cystic lesions (34 bronchogenic cysts, 15 pericardial cysts and 2 esophageal duplication cysts).

Based on the location of the centroid, it was possible to satisfactorily classify each mediastinal mass lesion into one compartment without any inconsistencies (Table II). Most tumors (77.1%, 343/445) were classified as being located in the anterior mediastinum, while 27 lesions (6.1%) were classified as being located in the superior portion of the mediastinum, 38 lesions (8.5%) in the middle mediastinum, and 37 lesions (8.3%) in the posterior mediastinum.

Almost all thymic epithelial tumors (99%, 244/246), all 24 thymic malignant lymphomas, and most germ cell neoplasms (93%, 54/58) were classified as being in the anterior mediastinum compartment. The majority of intrathoracic goiters (82%, 14/17) were categorized as being located in the superior portion of the mediastinum compartment. Of the remaining two goiters, one was located in the anterior and one was located in the middle mediastinum. Approximately two-thirds of mass lesions in the middle mediastinum were cysts, including foregut and pericardial cysts. On the other hand, two-thirds of pericardial cysts were located in the anterior mediastinum (particularly in the right cardiophrenic sinus). Approximately 80% of the 37 mass lesions in the posterior mediastinum were neurogenic tumors. Accordingly, 29 of the 49 neurogenic tumors (60%) were categorized as being located in the posterior mediastinum, while 10 (20%) were in the superior portion of the mediastinum, 4 (8%) were in the anterior mediastinum, and 6 (12%) were in the middle mediastinum.

## Discussion

In the present study, we described a new method for classifying the location of mediastinal lesions according to compartments seen on transverse plane images. We also investigated the use of this method in terms of its usefulness and user-friendliness. We found that this method has several strong features. First, a lesion’s compartment is defined as the compartment in which the lesion centroid is found, leaving little room for misclassification. Second, each compartment is clearly bound by drawing anatomical boundary reference lines. Finally, each compartment includes an original organ or structure from which the mediastinal lesion arose. Using this system, we were able to satisfactorily classify each of 455 mass lesions into one of the four compartments, and the compartment characteristics were considered to be useful in differential diagnoses.

Sone *et al* (15) assessed potential spaces of mediastinal compartments using a CT pneumomediastinography and described discrepancies between the anatomical classification method and Felson’s method (using a lateral chest radiograph) (4). Felson did not classify the mediastinum, but proposed a way to guess the location of a mass based on its location relative to two drawn lines. In Felson’s method, the boundary line between the anterior and middle mediastinum is drawn along the tracheal anterior edge and the cardiac posterior edge. At the center of the human body (i.e., the retrosternal space), the sagittal section plane shows the anterior mediastinal zone to be ahead of the anterior tracheal wall, the great vessels, and the pericardium. However, at the left side of the mediastinum, the posterior boundary of the anterior mediastinum spreads deeply along the aortic arch and left hilum, and this anatomical complexity produces inconsistency with the Felson’s method. Namely, the anterior compartment includes some masses possibly in both the anterior and middle mediastinum on a lateral chest radiograph. Except for this portion, the anterior mediastinum (under the superior portion of the mediastinum) in our presently described classification system corresponds to that in Felson’s method.

The anterior mediastinum contains the thymus, mediastinal fatty tissue and anterior mediastinal lymph nodes. Among these, the thymus is the most important organ in the anterior mediastinum since a majority of anterior mediastinal masses are tumors of thymic origin (8–11). After birth, the thymus is a bi-lobed, triangular gland that occupies the thyropericardiac space of the anterior mediastinum; it is located anterior to the proximal ascending aorta, the pulmonary outflow tract, and the superior vena cava, and it extends caudally sometimes down to the level of the diaphragm (8). Our proposed anterior mediastinum compartment corresponds to the normal location of the thymus. The great vessels (including the left brachiocephalic vein, superior vena cava, superior and inferior pulmonary veins, ascending aorta and the lateral rim of the aortic arch) may be pushed backward and downward by expanding neoplasms of thymic origin (e.g., thymic epithelial tumors and thymic malignant lymphomas) and germ cell tumors in the anterior mediastinal zone. These vascular structures are also barriers to prevent an anterior mediastinal tumor from extending into the posterior or inferior area. This is one reason why these structures are the anatomic posterior boundary of the anterior mediastinum.

Traditional anatomical method divides the mediastinum into two major compartments (the superior and inferior mediastinum) by an imaginary line extending from the sternal angle to the fourth intervertebral (Th4/5) disc. Felson (4) and Sone *et al* (15) stated that there is no need to classify the superior mediastinum due to the continuity of the inferior mediastinal zone. However, classifying the superior portion of the mediastinum has the advantage of making it easy to differentiate an intrathoracic goiter or neurogenic tumor of the thoracic inlet from other mediastinal tumors. We defined the boundary at the caudate rim of the brachiocephalic vein with the trachea (tracheal midline). This boundary is familiar to thoracic radiologists, physicians and surgeons, as it corresponds to an imaginary boundary of #2R (right upper paratracheal) and #4R (right lower paratracheal) mediastinal lymph nodes on the IASLC lymph node map (14). In the present study, the majority of intrathoracic goiters and one-fifth of neurogenic tumors were located in the superior portion of the mediastinum, and these tumors were the main type existing in this zone. Not classifying this compartment would impact differential diagnosis, in that many intrathoracic goiters would be categorized as being in the anterior or middle mediastinum.

Some previously described methods of mediastinal compartmentalization have stated that the heart and great vessels, trachea, and main bronchi are located in the middle mediastinum, while the esophagus is located in the posterior mediastinum (3,7,12). In contrast, we did not include the cardiovascular system in the JART General Rules for Study of Mediastinal Tumors (13). The anterior rims of great vessels, pericardium and heart are considered to be the posterior boundary of the anterior mediastinal compartment, while the posterior rims of the great vessels, pericardium and heart represent the anterior boundary of the middle mediastinal compartment. We decided that since the esophagus, trachea, and bronchi share an embryological origin (foregut, endoderm), these should all be classified into the same compartment (the middle mediastinum). This anatomical zone surrounds the tracheobronchoesophageal zone, and is similar to the ‘central zone’ described by Sone *et al* (15). The majority of the lesions found in the middle mediastinum included foregut cysts, tracheal and esophageal tumors, and lymphadenopathy.

It has not been clearly stated whether the paraaortic area surrounding the thoracic descending aorta is classified as part of the middle or posterior mediastinum. Our definition of this boundary is only the M-PBL (the transverse plane at 1 cm behind the anterior edge of vertebral body). Further classification techniques may be necessary due to the present lack of appropriate methods.

Methodologies that have classified the heart and great vessels as part of the middle mediastinum, have also defined the front boundary of the posterior mediastinum as the posterior cardiac edge (1,3,6,7). One methodology stated that the paravertebral region is bounded at the front by the anterior surface of the vertebral column and at the back by the chest wall (16). On the other hand, Felson’s method stated that the boundary of the middle mediastinum and posterior mediastinum is drawn along the line connecting a point on each thoracic vertebral body at 1 cm behind its anterior margin (4). We adopted this method as the anterior boundary for the posterior mediastinum. The posterior mediastinum corresponds to the paravertebral zone; however, there is no potential space in a normal situation as the paravertebral area is strongly surrounded by connective tissue, since air does not pass into this area during CT pneumomediastinography, Sone *et al* did not mention this compartment. However, this space becomes clearly evident when a mass lesion exists in this zone. Inclusion of this compartment is useful in diagnosing paravertebral neurogenic tumors using tumor location. Most neoplasms in the paravertebral zone are neurogenic tumors that arise from the dorsal root ganglion/neuron, most of which are located adjacent to the intervertebral foramen. This is one reason for setting the anterior boundary of the paravertebral zone at 1 cm behind the anterior margin of the vertebral body.

The literature does not include any previous descriptions of the posterior and lateral boundaries of the paravertebral zone. Therefore, based on expediency and experience, herein we set the bilateral posterolateral boundary lines of the paravertebral zone as bilateral vertical lines against the posterior rim of the chest wall at the lateral rim of the thoracic vertebral transverse process. However, in practice, it might be somewhat difficult to differentiate a paravertebral neurogenic tumor from an intercostal neurogenic tumor by using this boundary.

The present study had several limitations. First, it was a retrospective study, and there may have been a sample bias as a majority of the collected cases were anterior mediastinal masses. However, it is well known that anterior mediastinal masses occur with a higher incidence than those in other compartments (2,7–10). Second, we did not analyze inter- and intra-observer agreements for classifying masses into the four mediastinal compartments. Further study is needed to analyze reading agreements. Third, our proposal of this mediastinal compartment classification method has not yet been validated outside of Japan. The worldwide publication and review of this proposal will be important for validating this classification method for use in differential diagnosis of mediastinal masses in other populations.

In conclusion, we described a method for the mediastinum compartment classification based on transverse plane images obtained by CT, with the aim of providing a clinically practical method that can lead to more consistent and exact diagnosis of mediastinal masses. We also evaluated this novel classification method by using it to assess 445 mediastinal mass lesions. Our results indicate that the new mediastinal compartment classification method using CT transverse images is sufficiently user-friendly for practical clinical use and is potentially useful for the differential diagnoses of mediastinal mass lesions.

## Figures and Tables

**Table I:** Definition of the JART mediastinum compartments

Mediastinal compartment	Definition
Superior portion of the mediastinum	This compartment (area highlighted green in Figs. 1 and 2) is defined as the space between the superior border of the mediastinum (i.e., the thoracic inlet) and a horizontal plane at the intersection of the caudate margin of the brachiocephalic vein with the trachea [tracheal midline line (TML), black line in Figs. 1D and 2C]. This lower border is the same as the anatomical definition of the right upper paratracheal (#2R) lymph nodes, based on the lymph node map of the International Association for Study of Lung Cancer (IASLC) (14). The area is also anatomically bound anteriorly by the sternum, laterally by the parietal (mediastinal) pleural reflections, posteriorly by the anterior rim of the thoracic vertebral body; and posterolaterally by a vertical line against the posterior rim of the chest wall at the lateral rim of the thoracic vertebral transverse process (orange lines in figures).
Anterior mediastinum (Prevascular zone)	The anatomical boundaries of this compartment (area highlighted red in Figs. 1 and 2) are as follows: superior, the inferior boundary of the superior portion of the mediastinum (a horizontal line at the upper rim of the brachiocephalic vein where it ascends to the left, crossing in front of the trachea at the midline); inferior, the diaphragm; anterior, the sternum; lateral, the parietal (mediastinal) pleural reflections (including the lateral rims of the bilateral internal thoracic arteries and veins, and superior and inferior pulmonary veins); and posterior, the pericardium (including a horizontal line at the posterior rim of the heart), anterior rims of the left brachiocephalic vein, superior vena cava, superior and inferior pulmonary veins, ascending aorta, and the lateral rim of the aortic arch.
Middle mediastinum (Peritracheoesophageal zone)	The anatomical boundaries of this compartment (area highlighted yellow in Figs. 1 and 2) is anatomically bounded as follows: superior, the boundary of the superior portion of the mediastinum; inferior, the diaphragm; anterior, the posterior rim of the left brachiocephalic vein, superior vena cava, ascending aorta, bilateral main pulmonary arteries, and the heart; and posterior, the anterior rim of the descending aorta and a vertical line connecting a point on each thoracic vertebral body at 1 cm behind its anterior margin [middle-posterior boundary line (M-PBL), red lines in Figs. 1 and 2]. This compartment includes mainly the trachea, bilateral main bronchi and esophagus.
Posterior mediastinum (Paravertebral zone)	This compartment (area highlighted blue in Figs. 1 and 2) is anatomically bounded as follows: superior, the boundary of the superior portion of the mediastinum; inferior, the diaphragm; anterior, the boundary of the middle mediastinum (M-PBL); and posterio-lateral, a vertical line against the posterior rim of the chest wall at the lateral rim of the lateral process of the thoracic spine (orange lines in Figs. 1 and 2).

All descriptions of areas and lines in highlighted colors are illustrated in Figs. 1 and 2.

**Table II:** Result of the classification of the 445 mediastinal masses into the JART mediastinal compartment model.

	Mediastinal compartments				
		
Mediastinal masses	S	A	M	P	Total
Intrathoracic goiter	14	1	2	-	17
Thymoma	-	192	1	-	193
Thymic carcinoma	-	52	1	-	53
Thymic malignant lymphoma	-	24	-	-	24
Mature teratoma	2	24	-	1	27
Malignant germ cell tumors	-	30	1	-	31
Pericardial cyst	-	10	5	-	15
Bronchogenic cyst	1	6	20	7	34
Esophageal duplication cyst	-	-	2	-	2
Neurogenic tumors	10	4	6	29	49
Total	27	343	38	37	445

S, superior portion of the mediastinum; A, anterior mediastinum; M, middle mediastinum; P, posterior mediastinum.
